# In vivo assessment of myocardial viability after acute myocardial infarction: A head-to-head comparison of the perfusable tissue index by PET and delayed contrast-enhanced CMR

**DOI:** 10.1007/s12350-015-0329-7

**Published:** 2016-02-02

**Authors:** Stefan A. J. Timmer, Paul F. A. Teunissen, Ibrahim Danad, Lourens F. H. J. Robbers, Pieter G. H. M. Raijmakers, Robin Nijveldt, Albert C. van Rossum, Adriaan A. Lammertsma, Niels van Royen, Paul Knaapen

**Affiliations:** 1 Department of Cardiology, VU University Medical Center, De Boelelaan 1117, 5F013, 1081 HV Amsterdam, The Netherlands; 2 Department of Radiology & Nuclear Medicine, VU University Medical Center, Amsterdam, The Netherlands

**Keywords:** Infarction, myocardial, magnetic resonance imaging, myocardial viability, oxygen-15 water, myocardial perfusion imaging: PET

## Abstract

**Background:**

Early recognition of viable myocardium after acute myocardial infarction (AMI) is of clinical relevance, since affected segments have the potential of functional recovery. Delayed contrast-enhanced magnetic resonance imaging (DCE-CMR) has been validated extensively for the detection of viable myocardium. An alternative parameter for detecting viability is the perfusable tissue index (PTI), derived using [^15^O]H_2_O positron emission tomography (PET), which is inversely related to the extent of myocardial scar (non-perfusable tissue). The aim of the present study was to investigate the predictive value of PTI on recovery of LV function as compared to DCE-CMR in patients with AMI, after successful percutaneous coronary intervention (PCI).

**Methods:**

Thirty-eight patients with ST elevation myocardial infarction (STEMI) successfully treated by PCI were prospectively recruited. Subjects were examined 1 week and 3 months (mean follow-up time: 97 ± 10 days) after AMI using [^15^O]H_2_O PET and DCE-CMR to assess PTI, regional function and scar. Viability was defined as recovery of systolic wall thickening ≥3.0 mm at follow-up by use of CMR. A total of 588 segments were available for serial analysis.

**Results:**

At baseline, 180 segments were dysfunctional and exhibited DCE. Seventy-three (41%) of these dysfunctional segments showed full recovery during follow-up (viable), whereas 107 (59%) segments remained dysfunctional (nonviable). Baseline PTI of viable segments was 0.94 ± 0.09 and was significantly higher compared to nonviable segments (0.80 ± 0.13, *P* < .001). The optimal cut-off value for PTI was ≥0.85 with a sensitivity of 85% and specificity of 72%, and an area under the curve (AUC) of 0.82. In comparison, a cut-off value of <32% for the extent of DCE resulted in a sensitivity of 72% and a specificity of 69%, and an AUC of 0.75 (AUC PTI vs DCE *P* = .14).

**Conclusion:**

Assessment of myocardial viability shortly after reperfused AMI is feasible using PET. PET-derived PTI yields a good predictive value for the recovery of LV function in PCI-treated STEMI patients, in excellent agreement with DCE-CMR.

## Introduction

After an acute myocardial infarction (AMI), the injured myocardium contains both reversibly damaged (‘viable, or stunned’) and irreversibly damaged (‘non-viable’) tissue, even after successful restoration of coronary reperfusion. Early recognition of dysfunctional but viable myocardium is of clinical relevance, since affected segments have the potential of (complete) functional recovery. Of the various diagnostic techniques available for detecting viability in AMI, delayed contrast-enhanced cardiac magnetic resonance imaging (DCE-CMR) has been evaluated extensively, and it has been shown that the extent of regional hyperenhancement is inversely related to functional improvement after reperfusion.^1^,^2^ More recently, the presence of microvascular injury has been shown to have incremental value over DCE alone in predicting viability.^3^ Nonetheless, the significance of contrast patterns in AMI remains ambiguous, as other reports have shown differences in contrast wash-out due to ischemia-induced alterations in the pharmacokinetics of gadolinium.^4^,^5^ Consequently, dysfunctional but viable myocardium may also show hyperenhancement, thereby limiting the accuracy of DCE-CMR for delineating viable from necrotic myocardium in the (sub)acute phase of myocardial infarction.

An alternative method to detect myocardial viability is the perfusable tissue index (PTI), which is a positron emission tomography (PET)-derived parameter. PTI reflects the fraction of myocardium that is able to rapidly exchange water, i.e., that is perfusable by water.^6^ Consequently, differentiation between viable and non-viable myocardium is based on the concept that areas of necrotic tissue cannot exchange water rapidly. Recently, it was shown that parametric PTI images can be generated from a single PET-CT scan.^7^ Indeed, preliminary data indicate that PTI may be used as a predictor of functional recovery in AMI.^8^ The aim of the present study was to investigate the predictive value of PTI on recovery of LV function after successful primary PCI for AMI compared against a background of DCE-CMR.

## Materials and Methods

### Study Population

Thirty-eight consecutive patients with an acute ST elevation myocardial infarction (STEMI), presenting at the catheterization laboratory within 6 h after onset of symptoms and successfully treated by primary PCI (i.e., thrombolysis in myocardial infarction (TIMI) III flow after coronary stenting), were prospectively included in this study. STEMI was defined according to the European Society of Cardiology/ACCF/AHA/World Heart Federation Task Force for the Universal Definition of Myocardial Infarction as new ST elevation at the J point in at least 2 contiguous leads of ≥2 mm (0.2 mV) in men or ≥1.5 mm (0.15 mV) in women in leads V2-V3 and/or of ≥1 mm (0.1 mV) in other contiguous chest leads or the limb leads, in the absence of left ventricular (LV) hypertrophy or left bundle-branch block (LBBB).^9^ As described previously,^10^ all patients were treated according to the ESC guidelines for management of STEMI.^11^ Patients with three-vessel disease and those who were hemodynamically unstable were excluded, since repeat revascularization therapies were deemed probable during study follow-up. Other exclusion criteria were previous myocardial infarction or coronary revascularization procedures. Patients were examined 4-6 days and 3 months after the cardiac event with [^15^O]H_2_O PET and CMR. No adverse events occurred between primary PCI and the follow-up imaging sessions, and medication was kept constant between scans. The study was approved by the institutional review board, and all subjects signed an informed consent form within 24 h after PCI. The clinical trial was registered (http://www.trialregister.nl) under number NTR3164.

### PET Image Acquisition

[^15^O]H_2_O PET scans were acquired between 4 and 6 days and at 90 days after PCI using a Gemini TF-64 (Philips Healthcare, Best, The Netherlands) PET/CT scanner. [^15^O]H_2_O (370 MBq) was injected as a 5 mL bolus (0.8 mL·s^−1^) followed by a 35-mL saline chaser at a rate of 2 mL·s^−1^ with the simultaneous start of a 6-min dynamic scan sequence min. This scan was followed immediately by a low-dose (LD) CT scan during normal breathing to correct for attenuation (55 mAs; rotation time 1.5 s; pitch 0.825; collimation 64·0.625). The rate pressure product (RPP), being the product of heart rate and systolic blood pressure, was monitored during all PET studies.

### PET Image Analysis

All scans were checked for misalignment between LD CT and [^15^O]H_2_O scans, but none of the studies required corrections. All images were reconstructed using the three-dimensional row action maximum likelihood algorithm into 22 frames (1 × 10, 8 × 5, 4 × 10, 2 × 15, 3 × 20, 2 × 30, and 2 × 60 s) applying all appropriate corrections, i.e., normalization, dead time, decay, scatter, randoms, and attenuation based on the corresponding LD CT scan. Parametric PTI images were generated as previously described using the in-house developed software package *Cardiac VUer*.^7^ In brief, parametric images of perfusable tissue fraction (PTF), and arterial and venous blood volume fractions were calculated using a basis function implementation of the standard single tissue compartment model for [^15^O]H_2_O.^12^,^13^ Parametric images of arterial and venous blood volume fractions were subtracted from normalized CT transmission images, resulting in parametric anatomical tissue fraction (ATF) images. Parametric PTI images were calculated as the ratio of PTF and ATF. Finally, 16 myocardial volumes of interest (VOIs) were defined manually on parametric PTF images, according to the 16 segments model of the American Heart Association,^14^ after which this VOI template was projected onto the parametric PTI images. Furthermore, parametric myocardial blood flow (MBF) images were generated and quantitatively analyzed using Cardiac VUer. MBF was expressed in mL/min/g of perfusable tissue.

### CMR Image Acquisition

CMR was performed between 4 and 6 days and at 90 days after PCI using a 1.5 Tesla MR-scanner (Avanto, Siemens, Erlangen, Germany) with the use of a dedicated phased array cardiac receiver coil. Functional imaging was performed using retrospectively ECG-gated steady-state free precession cine imaging with breath holding. Standard 3 long axis orientations (4, 3, and 2 chamber views) and short axis orientation with full LV coverage were obtained (typical parameters: voxel size ~1.6 × 1.9 × 5.0 mm, slice thickness 5.0 mm, slice gap 5.0 mm, TR/TE 3.2/1.6 ms, flip angle 75º, field of view 360 × 400 mm, temporal resolution <50 ms). After administration of 0.2 mmol/kg gadolinium, DCE images were acquired after 10-15 min, using a 2-dimensional segmented inversion-recovery gradient-echo pulse sequence, with individual correction of the inversion time to null the signal of normal myocardium (slice thickness 5.0 mm, slice gap 5.0 mm, field of view 360 × 400 mm, pixel size ~1.4 × 1.4 mm, TR 2x RR interval, typical inversion time 250-350 ms). Cine and DCE images of each patient were matched by slice position.

### CMR Image Analysis

Analysis was performed with dedicated off-line software (QMassMR v7.5, Medis, Leiden, the Netherlands)^15^ Cine images were analyzed by tracing endocardial and epicardial myocardial borders in both end-diastolic and end-systolic phases. From these slices, myocardial volumes and ejection fraction were calculated. Left ventricular end-diastolic and end-systolic volumes were indexed for body surface area (LVEDVi and LVESVi, respectively)^16^ Systolic wall thickening (SWT) was calculated by subtracting end-diastolic from end-systolic wall thickness. Myocardial segments were considered to be dysfunctional if SWT was <3 mm, based on the mean SWT of 4.4 ± 0.7 mm (mean ± 2 SD) in a group of 10 healthy volunteers (age 50-75 years)^3^ Accordingly, viability was defined as complete recovery of systolic wall thickening (SWT) ≥3.0 mm at follow-up^3^,^17^ Quantification of infarct size and the size of the area containing microvascular injury (MVI) was performed on the short axis DCE images. CMR images were analyzed according to the 16-segment AHA model as used for the parametric PET images. The amount of DCE was calculated using the full-width at half-maximum method^18^ All areas of enhancement were quantified by computer-assisted planimetry on each of the short-axis images and the segmental extent of enhancement was expressed as a percentage of the segmental area. MVI was defined as a hypoenhanced area within the hyperenhanced infarcted myocardium^19^ MVI was included in the calculation of total infarct size. The extent of MVI was calculated for each patient, and expressed as the sum of the segments with MVI, as a percentage of the number of segments scored.

### Statistical Analysis

Continuous variables are presented as mean ± SD, and categorical data are summarized as frequencies and percentages. The significance of intra-individual differences between baseline and follow-up was assessed using the paired Student’s *t* test. Inter-individual differences between viable and non-viable myocardium were assessed using the unpaired Student’s t-test. Multiple datasets were compared using analysis of variance (ANOVA), and specific differences were identified using Student’s t-test with Bonferroni inequality adjustment. To identify independent predictors of LVEF at baseline and the change in LVEF between baseline and follow-up, multivariable linear regression analyses with a stepwise manual backward selection were applied and a removing probability for each variable of ≥0.1, and presented with standardized β-coefficients. Receiver operating characteristic (ROC) curves were generated for PTI, MBF, and DCE for the prediction of myocardial viability assessed by CMR. The area under the curve (AUC) was considered a measure of accuracy to discriminate between viable and non-viable myocardium. All statistical tests were 2 tailed, and a *P* value of ≤.05 was considered statistically significant. All statistical analyses were performed using the IBM SPSS software package (IBM SPSS Statistics 22, Chicago, IL, USA).

## Results

Baseline patient characteristics are listed in Table 1. None of the patients suffered from re-infarction, repeat revascularization, or hospitalization for heart failure between baseline and follow-up study.

**Table 1:** Baseline characteristics

Characteristic	AMI (*n* = 38)
Male sex	33 (87%)
Age (years)	58 ± 9
BMI (kg/m^2^)	27 ± 2
CAD risk factors	
Diabetes	1 (3%)
Hypertension	7 (18%)
Hypercholesterolemia	5 (13%)
Smoking history	29 (76%)
Family history	16 (42%)
Duration of symptoms (h)	1.7 ± 1.2
Time to reperfusion (h)	2.0 ± 1.2
CK-MB peak (U/L)	180 ± 197
Infarct-related artery	
LAD	21 (55%)
RCx	3 (8%)
RCA	14 (37%)
TIMI-3 flow grade after PCI	35 (92%)

*AMI*, acute myocardial infarction; *BMI*, body mass index; *CAD*, coronary artery disease; *CK-MB*, creatine kinase-MB; *LAD*, left anterior descending; *RCx*, ramus circumflex; *RCA*, right coronary artery; *TIMI*, thrombolysis in myocardial infarction

### Global Analysis

There was no significant difference between LVEF at baseline and follow-up (i.e., 50.1 ± 7.3% vs 51.0 ± 8.4%, *P* = .34). LVEDVi and LVESVi averaged 92 ± 12 and 47 ± 12 mL/m^2^ at baseline, which were not significantly different at follow-up (i.e., 95 ± 19 mL/m^2^, *P* = .29, and 48 ± 18 mL/m^2^, *P* = .86, respectively). DCE averaged 20 ± 12% of total LV mass at baseline, which was significantly reduced at follow-up (12 ± 7%, *P* < .001). MVI was present in 22/38 (58%) of patients at baseline, and in none at follow-up. When present, MVI affected an average of 5 ± 2 segments (out of 16, i.e., 31 ± 14%) per patient.

PTI at baseline was 0.87 ± 0.06 and was not significantly different at follow-up (0.88 ± 0.05, *P* = .59). PTF and ATF did also not change from baseline to follow-up, i.e., from 0.66 ± 0.06 to 0.65 ± 0.05 (*P* = .27) and from 0.76 ± 0.05 to 0.75 ± 0.03 (*P* = .09), respectively. There was a significant reduction in resting MBF between baseline and follow-up, changing from 0.97 ± 0.22 to 0.87 ± 0.15 mL/min/g (*P* = .01). The heart rate dropped from 66 ± 11 to 62 ± 10 b/pm (*P* = .02) between baseline and follow-up. Systolic blood pressure increased from 106 ± 15 to 111 ± 11 mmHg (*P* = .01), whereas diastolic blood pressure remained unchanged (59 ± 8 mmHg vs 62 ± 8, *P* = .20). Overall, the RPP was not significantly different between PET-studies (7019 ± 1741 vs 6946 ± 1484 mmHg/min, *P* = .85), indicating comparable hemodynamic conditions.

### Regional Analysis

A total of 588 (97%) out of 608 segments were available for serial analysis. Twenty segments were excluded based on insufficient quality. A total of 331 remote segments were normokinetic at baseline and showed no contrast enhancement. These segments were defined as remote myocardium. A total of 257 segments showed SWT of less than 3 mm, of which 180 (70%) exhibited DCE and were located in the myocardial territory of the culprit-artery. All patients had dysfunctional segments with DCE, with an average of 5 ± 3 segments per patient. These latter segments were defined as infarcted myocardium.

#### Infarcted vs remote myocardium

PTI was significantly reduced in infarcted vs remote myocardium (0.86 ± 0.14 vs 0.94 ± 0.10, *P* = .001). Resting MBF averaged 0.93 ± 0.21 mL/min/g in the infarcted myocardium vs 0.96 ± 0.23 mL/min/g in remote myocardium (*P* = .58). SWT was severely depressed in infarcted myocardium compared with remote myocardium (1.4 ± 1.1 vs 4.1 ± 1.0 mm, *P* < .001). Additional baseline PET and CMR data, subdivided per quartile of infarct transmurality, are summarized in Table 2.

**Table 2:** Segmental PET and CMR data at baseline

	Extent of DCE (%)				
	Remote	1–25 (*n* = 63)	26–50 (*n* = 58)	51–75 (*n* = 34)	>75 (*n* = 25)
PTI*	0.94 ± 0.10	0.90 ± 0.12	0.87 ± 0.13**	0.82 ± 0.13**	0.77 ± 0.15**
PTF*	0.71 ± 0.10	0.67 ± 0.09	0.66 ± 0.09	0.63 ± 0.08**	0.60 ± 0.10**
ATF	0.77 ± 0.06	0.75 ± 0.09	0.77 ± 0.09	0.77 ± 0.08	0.82 ± 0.07
MBF* (mL/min/g)	0.96 ± 0.23	0.96 ± 0.22	0.95 ± 0.22	0.86 ± 0.20	0.81 ± 0.21**
MVI*	n.a.	16/63 (25%)	20/58 (35%)	14/34 (41%)	19/25 (75%)
Viable* (%)	n.a.	4/63 (70%)	19/58 (33%)	5/34 (14%)	5/25 (20%)

*DCE*, delayed contrast enhancement; *PTI*, perfusable tissue index; *PTF*, perfusable tissue fraction; *ATF*, anatomical tissue fraction; *MBF*, myocardial blood flow; *MVI*, microvascular injury; *n.a.*, not applicable

*P < .01 (ANOVA), **P < .01 vs remote

At follow-up, PTI, PTF, and ATF of infarcted myocardium remained unchanged (0.88 ± 0.14, 0.64 ± 0.09, 0.74 ± 0.09, all *P* = NS vs baseline). Resting MBF in infarcted myocardium, however, was significantly reduced compared with remote myocardium (0.80 ± 0.19 vs 0.88 ± 0.15 mL/min/g, *P* = .05). SWT of infarcted myocardium significantly improved to 2.3 ± 1.7 mm (*P* < .001 vs baseline). Follow-up PTI, PTF, ATF, DCE, and SWT data for remote myocardium were all comparable to baseline values (data not shown).

#### Viable vs non-viable

Of the 180 infarct-related myocardial segments, 73 showed recovery during follow-up and were classified as viable (i.e., stunned myocardium), whereas 107 segments remained dysfunctional and were classified as non-viable. Baseline PTI of viable segments was 0.94 ± 0.09, which was significantly higher than that of non-viable segments (0.80 ± 0.13, *P* < .001). The extent of DCE at baseline averaged 26 ± 24% in viable segments vs 59 ± 27% in non-viable segments (*P* < .001).

**Figure 1:**
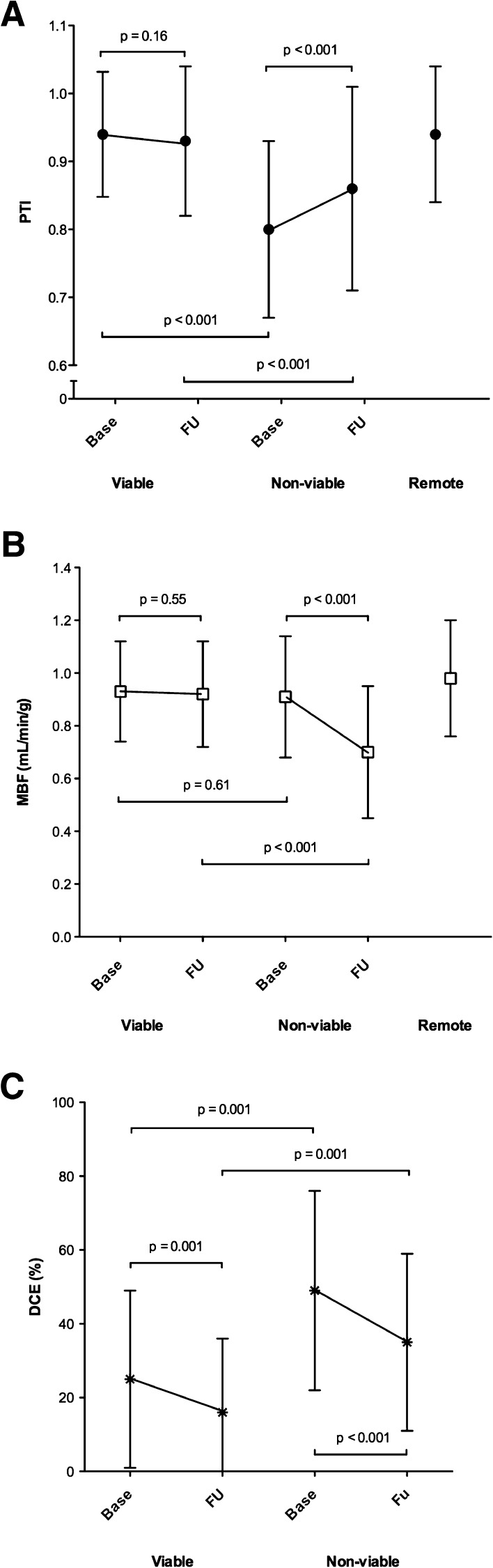
Serial changes in **(A**) PTI, (**B**) MBF and (**C**) DCE for viable and non-viable myocardial segments from baseline to follow-up, in relation to remote myocardium. *PTI*, perfusable tissue index; *MBF*, myocardial blood flow; *DCE*, delayed contrast enhancement; *Base*, baseline; *FU*, follow-up

SWT of viable segments improved from 1.8 ± 1.0 to 4.0 ± 1.0 mm (*P* < .001), whereas SWT of non-viable segments did not change (1.2 ± 1.1 mm vs 1.1 ± 1.0, *P* = .70). Correspondingly, MBF in viable segments was preserved at follow-up (0.91 ± 0.23 mL/min/g), whereas MBF in non-viable segments was reduced to 0.70 ± 0.25 mL/min/g (*P* < .001 vs baseline). There was a significant difference between the presence of MVI at baseline in viable and non-viable segments (29% vs 45%, *P* < .001). Figure 1 illustrates the evolution of PTI, MBF, and DCE for viable and non-viable myocardium over time, in relation to remote myocardium.

### Prediction of Function and Recovery

#### Regional recovery

**Figure 2:**
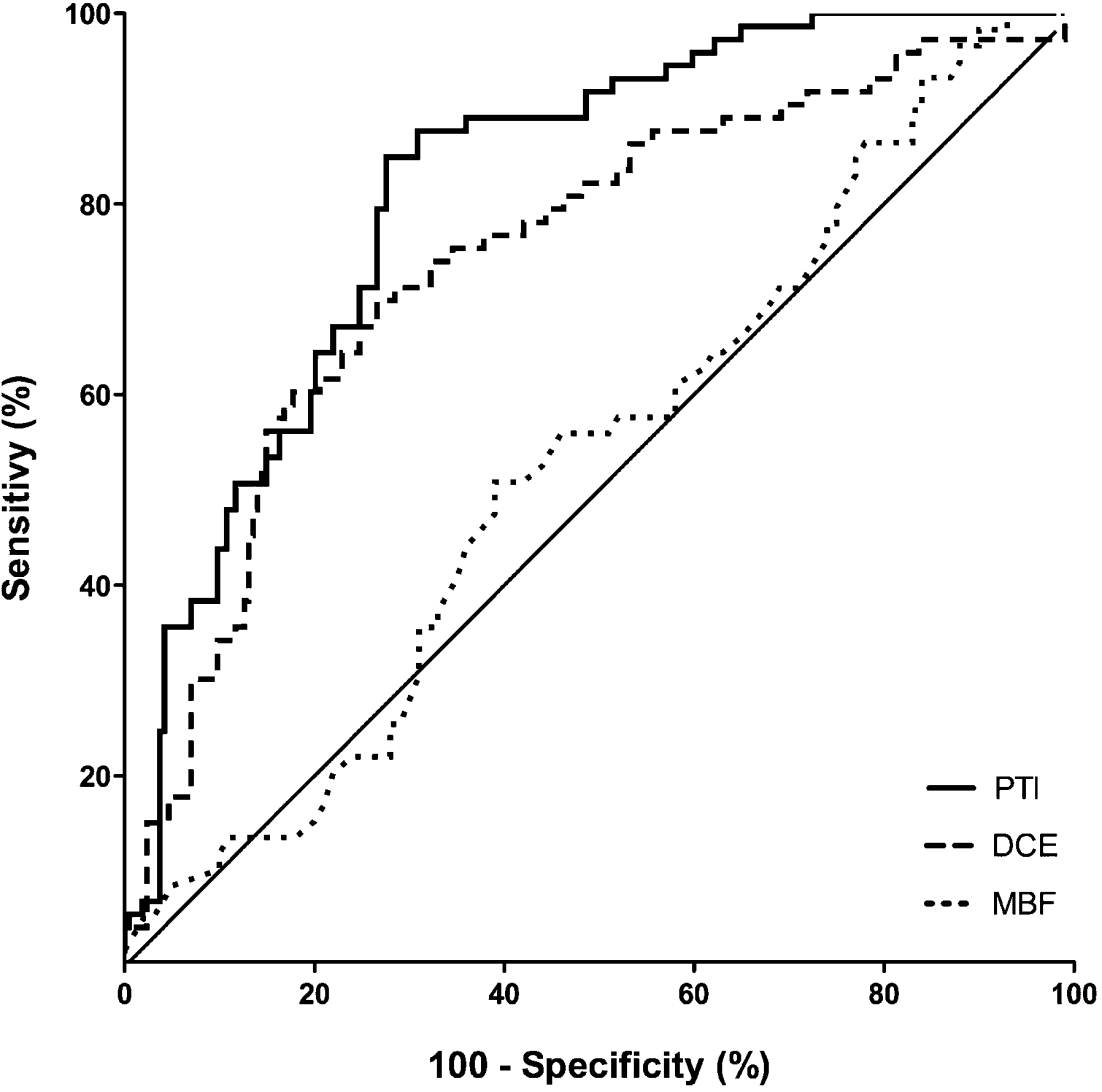
PTI, DCE, and MBF receiver operator characteristics curves for differentiating between viable and non-viable segments. *PTI*, perfusable tissue index; *DCE*, delayed contrast enhancement; *MBF*, myocardial blood flow

**Figure 3:**
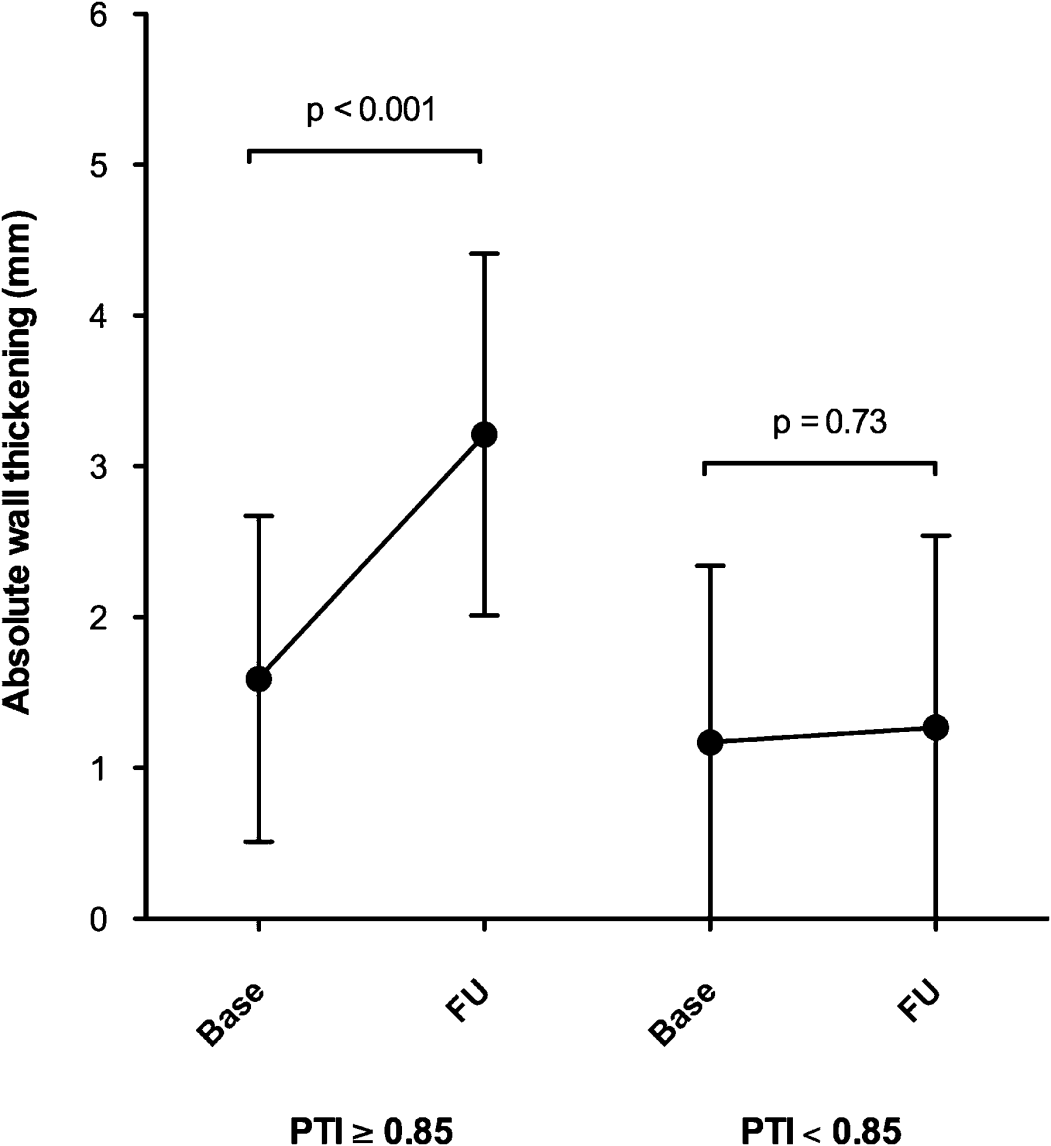
Serial changes in regional function from baseline to follow-up in segments in the territory of the culprit artery, subdivided for PTI ≥0.85 and PTI <0.85. *PTI*, perfusable tissue index; *Base*, baseline; *FU*, follow-up

Figure 2 depicts the values of PTI, DCE, and MBF for predicting myocardial viability (PTI: AUC 0.82, CI 0.76-0.88; DCE: AUC 0.75, CI 0.67-0.82; resting MBF: AUC 0.53, CI 0.44-0.62). The AUC was not significantly different between PTI and DCE (*P* = .14). The optimal cut-off value for the PTI was ≥0.85, yielding a sensitivity, specificity, positive predictive value (PPV) and negative predictive value (NPV) of 85%, 72%, 67%, and 88%, respectively. In comparison, a cut-off value of <32% for the extent of segmental DCE resulted in a sensitivity of 72% and a specificity of 69%, with a PPV of 58% and a NPV of 80%. Figure 3 illustrates the baseline and follow-up data for regional function in the myocardial territory of the culprit artery, subdivided for segments with a PTI ≥0.85 and <0.85.

#### Global function and recovery

Table 3 demonstrates univariable and multivariable linear regression analyses for the prediction of baseline LVEF and the change in LVEF between baseline and follow-up. PTI ≥0.85, infarct size, presence and extent of MVI were all predictors of baseline LVEF. Multivariable analysis revealed that the extent of MVI was the strongest and single independent predictor of baseline LVEF (β = −0.69; *P* < .001). For the change in LVEF at follow-up, PTI ≥0.85 was the only significant predictor (β = 0.34; *P* = .04).

**Table 3:** Univariable and multivariable regression analysis for the prediction of baseline LVEF and the absolute change in LVEF between baseline and follow-up (Δ LVEF, per %)

Variable	LVEF at baseline				Δ LVEF	
	Univariable		Multivariable		Univariable	
	β	*P* value	β	*P* value	β	*P* value
PTI ≥0.85	0.50	0.002			0.34	0.04
MBF (mL/min/g)	0.13	0.34			−0.11	0.51
Infarct size (% LV)	−0.54	<0.001			−0.16	0.34
Presence of MVI	−0.54	<0.001			−0.18	0.27
Extent of MVI (% LV)	−0.65	<0.001	−0.69	<0.001	−0.12	0.46

*LVEF*, left ventricular ejection fraction; *LV*, left ventricle; other abbreviations as in Table 2

**Figure 4:**
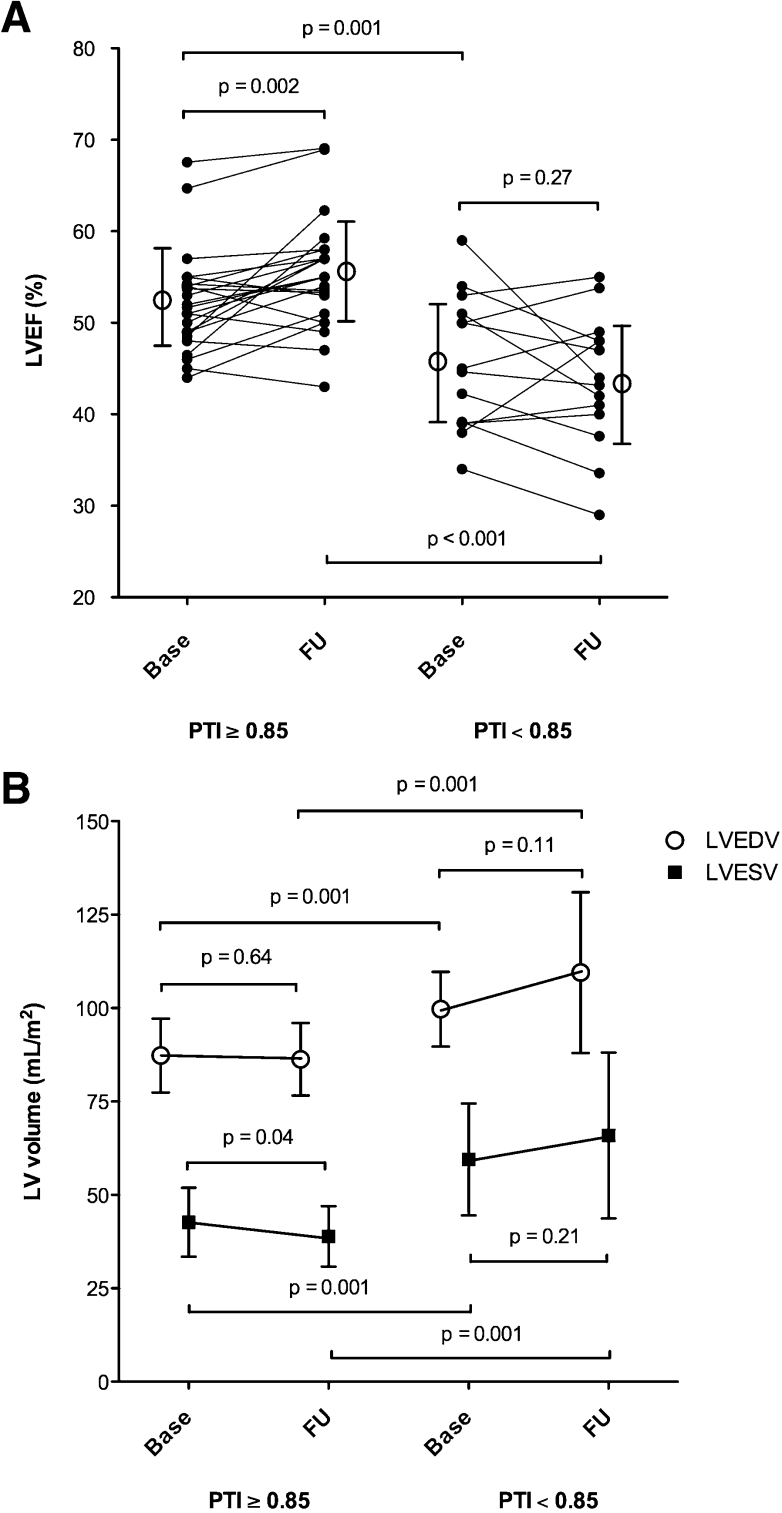
Serial changes in (**A**) LVEF and (**B**) global left ventricular volumes from baseline to follow-up in patients with viable (PTI ≥0.85) and non-viable myocardium (PTI <0.85). *LVEF*, left ventricular ejection fraction; *LVEDV*, left ventricular end-diastolic volume index; *LVESV*, left ventricular end-systolic volume index; *PTI*, perfusable tissue index; *Base*, baseline; *FU*, follow-up

**Figure 5:**
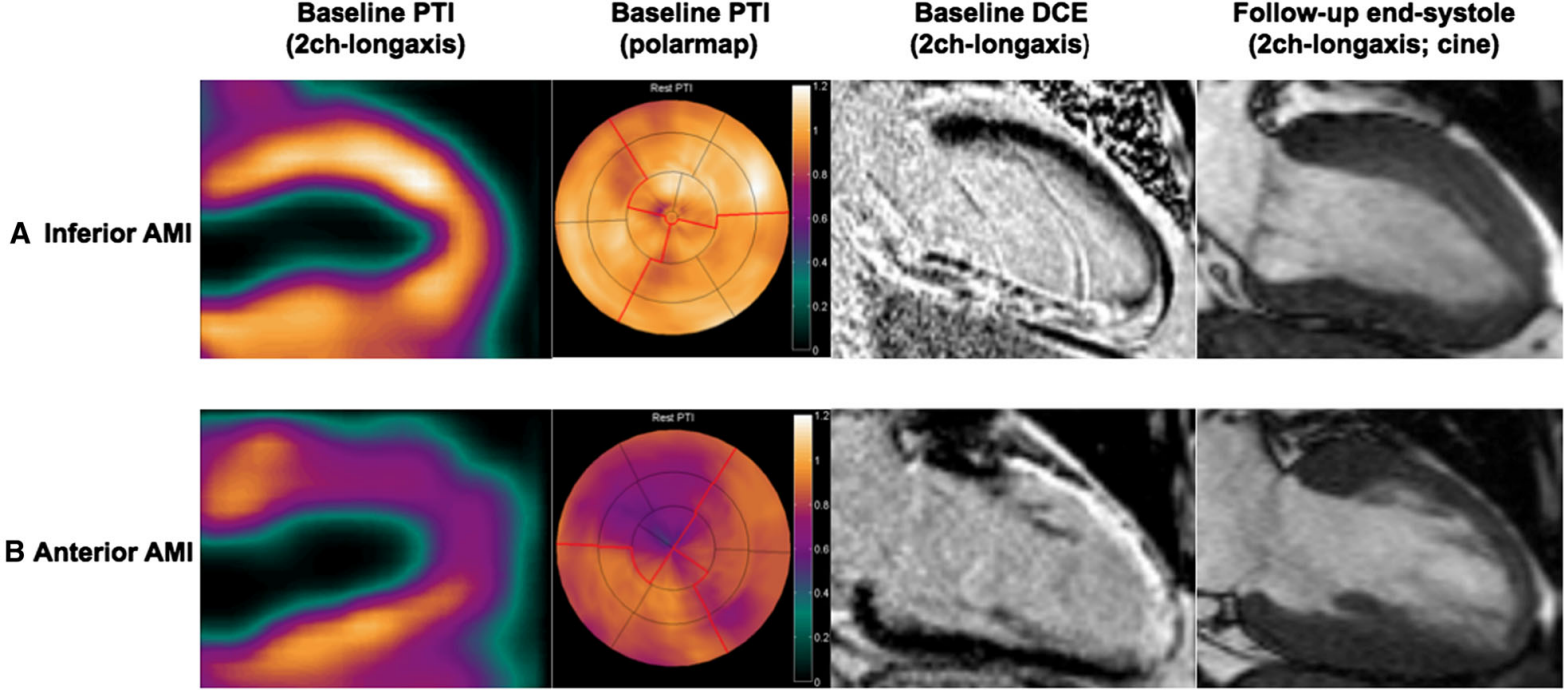
Two patients with AMI after successful reperfusion both showing extensive DCE; (**A**) Inferior AMI with preserved PTI of the inferior wall and functional recovery at follow-up. (**B)** Anterior AMI with reduced PTI of the anterior wall and no functional recovery at follow-up. *AMI*, acute myocardial infarction; *DCE*, delayed contrast enhancement; *PTI*, perfusable tissue index

Figure 4 illustrates the baseline and follow-up data for LVEF and LV volumes subdivided for patients with viable vs non-viable myocardium, when applying the PTI cut-off value of 0.85. Overall, an increase in LVEF was observed in patients with PTI ≥0.85 (*P* = .002), as well as a preservation of LVEDVi (*P* = .64), and a reduction of LVESVi (*P* = .04). In patients with PTI <0.85, no recovery in LVEF, LVEDVi, and LVESVi were seen. 33% of patients with PTI ≥0.85 (8 out of 24) showed an increase in LVEF of at least 5%, vs none in the patients with PTI <0.85. Figure 5 illustrates the functional recovery by CMR of two study subjects, in relation to baseline PTI and DCE.

## Discussion

The main finding of this study is that PTI can identify viable myocardium in patients with AMI following successful revascularization with fairly good diagnostic accuracy in absolute terms, and in excellent agreement with DCE-CMR. On a segmental level, a PTI cutoff value of 0.85 yielded the best diagnostic accuracy for discriminating between viable and non-viable myocardium. On a global level, a PTI ≥0.85 in infarcted myocardium was associated with an improvement in LVEF.

### PTI as a Marker of Viability

PTI reflects the fraction of extravascular myocardial tissue that can rapidly exchange water (i.e., PTF), in relation to its anatomic counterpart ATF. The likelihood of functional recovery of stunned myocardium depends on the degree of irreversible damage (i.e., tissue necrosis). Assuming that necrotic tissue does not exchange water, the main determinant of PTI is viable (perfusable) myocardium. Consequently, dysfunctional segments with a normal or near normal PTI are expected to be viable because of a limited amount of myocardial damage. In contrast, dysfunctional myocardium with a reduced PTI is less likely to be viable because more necrotic tissue is present. In the subacute phase after reperfused AMI, PTI in viable segments was not significantly different from remote control segments. In contrast, PTI was significantly reduced in the non-viable segments as compared with the remote segments.

The predictive value of PTI in AMI has been studied previously by Yamamoto et al^8^ In that study, PTI was determined in 11 patients who were successfully treated with thrombolysis following AMI. Of 12 dysfunctional segments at baseline, 7 showed improved systolic wall thickening at follow-up, as measured by echocardiography. PTI in segments that recovered averaged 0.88, which is only slightly lower than the values seen in the present study. Furthermore, PTI in viable segments was not significantly different from remote segments, in accordance with the present findings. Follow-up time in the present study (97 ± 10 days) was comparable to the study by Yamamoto et al. and was shown to be valid for detection of myocardial recovery after myocardial infarction.^8^

Complete recovery of function only occurred in segments with a PTI >0.80, suggesting that at least 80% of a myocardial segment needs to survive after AMI, in order for it to regain function. Previous investigations in patients undergoing revascularization therapy for chronic MI have reported similar preconditions for tissue viability, ranging from 0.70 to 0.80 for the PTI.^20^-^22^ Although PTI in non-viable segments averaged 0.80, it should be noted that a small number of non-viable segments exhibited higher values (up to 0.99), indicating a certain degree of overlap in PTI values between viable and non-viable myocardium. This is, at least in part, due to the fact that in the present study viability is expressed in a binary (i.e. segments are graded either viable or non-viable) rather than a gradual fashion, where the latter is likely to be a more accurate reflection of reality^23^ Indeed, non-viable segments with PTI >0.80 showed some functional recovery at follow-up, averaging 1.0 mm, whereas non-viable segments with PTI <0.80 showed no recovery at all, or even exhibited dyskinesia.

### PTI vs DCE in (Sub)Acute MI

Although the extent of hyperenhancement, as assessed by CMR, was accompanied by a gradual decrease in PTI and PTF (in line with the study hypothesis), a systematic discrepancy was observed between the extent of DCE and PTI with increasing infarct size. DCE-CMR has been investigated and validated extensively for the detection of myocardial viability in chronic MI.^24^,^25^ Experimental studies have shown that the extracellular contrast agent gadolinium only accumulates in irreversibly damaged tissue, thereby providing accurate delineation of non-viable from viable tissue.^26^,^27^ The significance of contrast patterns in acute MI, however, is less clear and, despite extensive research, results are not unambiguous. In contrast to chronic MI, which histologically is based on fibrosis and scar tissue, (sub)acute MI causes formation of tissue edema and/or eventual disruption of the myocyte membrane, altering wash-in/wash-out kinetics of gadolinium and increasing its volume of distribution as well, albeit on a different pathophysiological basis.^28^ In addition, on histological examination, even in so-called transmural myocardial infarction, viable islets of cardiomyocytes can frequently be detected within the scar region.^29^ Hence, areas that contain a considerable amount of viable myocardium may still show hyperenhancement, as the signal will be dominated by the (possibly small) fraction of transmural necrosis.^30^ The physiological consequence may be that actual infarct size is overestimated with DCE-CMR, as hyperenhanced areas may be composed of a mixture of necrotic tissue and, to a lesser extent, reversibly damaged, edematous tissue, predominantly in the setting of (sub)acute MI. This is highlighted by the fact that 5/25 (20%) of myocardial segments with (near) transmural infarction (≥75% hyperenhancement) actually recovered completely during follow-up. These numbers are in accordance with previous reports and imply that contrast enhancement is not merely limited to areas of tissue necrosis, but also occurs in myocardial tissue with non-critical injuries. The fact that PTI in these recovered segments averaged 0.98 (vs 0.72 in non-viable segments, *P* < .001) emphasizes that the main parts of these segments are capable of rapidly exchanging water despite extensive hyperenhancement. Under these circumstances, DCE-CMR may potentially cause erroneous interpretation of tissue viability.^31^ The latter is illustrated in Fig. 4, which depicts the parametric PTI and (DCE-)CMR images of two study subjects with extensive hyperenhancement after successfully reperfused AMI. The patient with an inferior wall AMI (Fig. 4A) exhibits a near normal PTI, whereas the patient with an anterior wall AMI (Fig. 4B) shows a severely reduced PTI. Although both patients exhibit transmural contrast enhancement of the infarcted area, functional recovery was only observed in the infarcted areas with preserved PTI, suggesting that contrast enhancement may not reliably differentiate between reversibly damaged (e.g. edematous) and irreversibly damaged (e.g. necrotic) myocardium.

### Temporal Infarct Evolution

Whereas PTI in viable myocardium did not significantly change during follow-up, PTI in non-viable myocardium significantly increased (albeit limited), mainly due to a reduction in ATF. This reduction in ATF may be the result of partial volume effects caused by wall thinning, which was more apparent in the non-viable myocardial segments. At follow-up, resting myocardial perfusion was significantly decreased in non-viable myocardium as opposed to viable myocardium. This can be related to a severe reduction in contractility due to extensive loss of cardiomyocytes, which results in a reduction of tissue oxygen demand, and thus MBF. Considering that contractility recovered in viable myocardium, metabolic demand is maintained in these segments during infarct evolution. Finally, a significant reduction in hyperenhancement was seen between baseline and follow-up, for both viable and non-viable myocardium, mimicking a reduction in infarct size. This may be attributed to several reasons mentioned previously, being (1) overestimation of infarct size in the (sub)acute phase of AMI as a result of ischemia induced alterations in wash-in/wash-out kinetics of gadolinium and volume of distribution, and (2) infarct shrinkage during follow-up due to the replacement of necrotic tissue by collagenous scar, resulting in a denser, yet smaller tissue volume.

### Microvascular Injury

MVI is characterized by extensive damage to the microcirculation resulting in severely impaired tissue perfusion, and previous studies have reported MVI as a powerful predictor of long-term outcome^32^ and functional recovery^2^,^3^ in patients with reperfused AMI. Indeed, MVI was associated with a reduced LVEF at baseline and the extent of MVI was the only independent predictor of baseline function, as previously reported.^3^ Contrary to previous observations however, there was no significant relationship between baseline MVI and functional recovery at a global level, although there was significantly less MVI in viable segments. This apparent discrepancy between global and segmental findings may be attributed to the fact that approximately one third of all viable segments actually exhibited a certain degree of MVI.

### Clinical Implications

The high NPV of PTI enables the use of PET to rule out viability in dysfunctional segments after acute MI, without the need for traditional nuclear metabolic imaging. PTI can be obtained in less than 10 min and radiation burden is low. The limited availability of PET scanners worldwide and the need for an on-site cyclotron, however, hampers its clinical applicability and currently favor the use of alternative imaging techniques, such as CMR, for assessing tissue viability in patients with acute/chronic MI.

### Methodological Considerations

Several methodological aspects should be taking into consideration. First, the use of absolute wall thickening as a means of measuring functional recovery does not account for potential tethering of non-viable segments to surrounding viable segments, and may falsely give the impression of improved function.^23^ Second, during infarct evolution the spatial extent of hyperenhancement on CMR is reduced, as the acutely necrotic core is replaced by collagenous scar. This results in shrinkage of the infarct and may introduce a certain degree of misalignment of myocardial segments.^26^ Although this may have introduced bias in favor of CMR, i.e. CMR was used to assess both wall motion and DCE, the effect is expected to be limited since ATF of the infarcted myocardium was comparable between baseline and follow-up. Similarly, results from different imaging modalities were combined and serial analysis of myocardial segments was performed. Despite the fact that care was taken in matching myocardial territories, some misalignment might have occurred. Finally, incorporation of a dobutamine stress protocol during CMR could have further enhanced delineation between viable and nonviable myocardial segments based on contractile reserve.

## Conclusion

Assessment of myocardial viability shortly after reperfused AMI is feasible using PET. PET derived PTI is a fairly good prognostic indicator for recovery of myocardial function, in excellent agreement with DCE-CMR.

## New Knowledge Gained

- PTI can identify viable myocardium in patients with AMI following successful revascularization with fairly good diagnostic accuracy in absolute terms, in excellent agreement with DCE-CMR.
- On a segmental level, a PTI cutoff value of 0.85 yielded the best diagnostic accuracy for discriminating between viable and non-viable myocardium.
- On a global level, a PTI ≥0.85 in infarcted myocardium was associated with an improvement in LVEF.

## Abbreviations

AMI: Acute myocardial infarction
CMR: Cardiovascular magnetic resonance
LAD: Left anterior descending artery
LCX: Left circumflex artery
LGE: Late gadolinium enhancement
LVEF: Left ventricular ejection fraction
PCI: Percutaneous coronary intervention
PTI: Perfusable tissue index
RCA: Right coronary artery
STEMI: ST-segment elevation myocardial infarction
